# *De Novo* Transcriptome Assembly and Differential Gene Expression Profiling of Three *Capra hircus* Skin Types during Anagen of the Hair Growth Cycle

**DOI:** 10.1155/2013/269191

**Published:** 2013-05-20

**Authors:** Teng Xu, Xudong Guo, Hui Wang, Xiaoyuan Du, Xiaoyu Gao, Dongjun Liu

**Affiliations:** The Key Laboratory of Mammalian Reproductive Biology and Biotechnology of the Ministry of Education, Inner Mongolia University, Hohhot, Inner Mongolia Autonomous Region 010021, China

## Abstract

Despite that goat is one of the best nonmodel systems for villus growth studies and hair biology, limited gene resources associated with skin or hair follicles are available. In the present study, using Illumina/Solexa sequencing technology, we *de novo* assembled 130 million mRNA-Seq reads into a total of 49,115 contigs. Searching public databases revealed that about 45% of the total contigs can be annotated as known proteins, indicating that some of the assembled contigs may have previously uncharacterized functions. Functional classification by KOG and GO showed that activities associated with metabolism are predominant in goat skin during anagen phase. Many signaling pathways was also created based on the mapping of assembled contigs to the KEGG pathway database, some of which have been previously demonstrated to have diverse roles in hair follicle and hair shaft formation. Furthermore, gene expression profiling of three skin types identified ~6,300 transcript-derived contigs that are differentially expressed. These genes mainly enriched in the functional cluster associated with cell cycle and cell division. The large contig catalogue as well as the genes which were differentially expressed in different skin types provide valuable candidates for further characterization of gene functions.

## 1. Introduction

Inner Mongolia Cashmere Goat (*Capra hircus*, IMCG) is a diploid (2*n* = 60) mammal that belongs to the family of *Bovidae*. It plays an important role in the world animal fiber industry because it can produce high quality underhair (cashmere is the commercial name) and is one of the world's largest breeding groups. Cashmere produced by IMCG, which is of a small diameter (14–18 *μ*m) and is soft to touch, is grown from the secondary hair follicle (HF) of the body skin [1, 2]. Fiber diameter and length determine both the quality and the amount of cashmere produced by an animal. The longer the length and the smaller the diameter of the cashmere fibers, the higher the price becomes. IMCGs exhibit seasonal rhythm and annual cycle of cashmere growth that are controlled by daylength. During the period from the summer solstice to midwinter, when the length of day is reduced, cashmere fiber has a high growth speed; in contrast, it becomes low during the period from midwinter to the next summer solstice [3, 4]. This photoperiodic characteristic of cashmere fiber growth is convenient for cashmere harvest and formulating a management strategy of cashmere production. During the past decades, many mammalian genomic and transcript sequences have become available, including *Homo sapiens*, *Mus musculus,* and *Bos taurus* which play important roles in understanding HF formation and hair growth. However, only a total of 561 ESTs are pertinent to goat skin or hair follicle indicating that few studies focused on understanding the gene expression pattern of goat skin or hair follicle. On the other hand, almost all the deposited goat skin associated ESTs were sequenced by the traditional approach from randomly picked cDNA clones which did not guarantee that the less-abundant transcripts could be efficiently detected. In addition, we also observed that cashmere fibers of IMCG are mainly produced by the back (BK) and side of the body (BS) skin of the trunk coat, but few are produced by the belly (BL) skin. This indicated that the gene expression patterns of BK and BS skin are different from BL. Therefore, the gene expression patterns and differential gene expression profiling of three skin sites during active hair growth phase (anagen) are important to understanding the underlying molecular mechanism associated with cashmere fiber growth. 

The advents of the ultra-high-throughput, cost-effective next-generation sequencing (NGS) technologies make the whole transcriptome sequencing and analysis feasible, even in the absence of genomic data [5]. In the past few years, NGS has been widely used in RNA sequencing (RNA-Seq) which provided researchers with more information about gene expression, regulation, and networks under specific physiological conditions or developmental stages in both model and nonmodel organisms [6–8]. It offers accurate quantitative and digital gene expression profiles of sequenced transcripts [5]; moreover, it has very low background noise and a large dynamic range of gene expression levels compared with DNA microarray [5]. In the present study, we utilized Illumina/Solexa paired-end mRNA-Seq approach to sequence and *de novo* assemble the goat skin transcriptome during anagen phase, and further investigated the gene expression profiles of three different skin types based on the assembled contigs, skin from the BL, BK, and BS of the trunk coat, which have a large discrepancy in cashmere production. 

## 2. Materials and Methods

### 2.1. Goat Skin Tissue Collection and RNA Extraction

Generally, according to the different skin types of trunk coat, skin from the back and body side of IMCG produce either wool or cashmere, whereas belly skin produces mainly wool fiber and few cashmere [9]. A breeding, two-year-old female goat was sampled from Yi Wei White Cashmere Goat Breeding Farm at Ulan Town of Erdos in Inner Mongolia Autonomous Region, China. During anagen phase (Nov, 2010), the hair (wool and cashmere) of belly, back, and body sides of the goat were sheared and further shaved. After sterilization with 70% alcohol, full-thickness skin sections of each body part were excised and immediately frozen in liquid nitrogen for storage and transport until RNA isolation. Goat skin tissue collections were carried out in accordance with the guidelines of Inner Mongolian Animal Society Ethics Committee. This study has been checked and approved by Inner Mongolian Animal Society which is responsible for Animal Care and Use in Inner Mongolia Autonomous Region, China. Skin excision was performed after Xylazine hydrochloride anesthesia, and all efforts were made to minimize suffering. Before RNA extraction, each sample was washed with 10 mL PBS (pH 7.2) and 0.5 mM EDTA. Total RNA of each sample was isolated by using a TRIzol plus RNA purification kit according to the manufacturer's protocol (Invitrogen). Total RNA quality and concentration were determined by a 2100 bioanalyzer nanochip (Agilent). Enrichment of mRNA from total RNA was performed with a RiboMinus RNA-Seq Kit according to the manufacturer's instructions (Invitrogen). 

### 2.2. Paired-End Library Construction and Illumina Sequencing

It has been reported that mRNA fragmentation can result in a more even coverage along the entire gene body, whereas cDNA fragmentation is more biased towards the 3′ end of the transcript [8]. Therefore, each ploy(A)-enriched RNA sample was chemically fragmented into small pieces by using divalent cations at 94°C for 5 min. The fragmented RNA was reverse-transcribed into cDNA by using random hexamer primers containing a tagging sequence at the 3′ end with the use of a superscript III double-stranded cDNA synthesis kit according to the manufacturer's protocol (Invitrogen). The double-stranded cDNA was subjected to end-repair and further 3′ terminal tagging by the addition of 5′ DNA adaptors and T4 DNA ligase with overnight incubation at 16°C for 16 hours. The targeted di-tagged cDNA was purified by polyacrylamide gel electrophoresis (PAGE) and gel excision (200 ± 25 bp). The clean di-tagged cDNA was enriched by limited-cycle PCR amplification (18 cycles) with primer pairs that annealed to the tagging sequences of the di-tagged cDNA. Library purification by PAGE removed any residual nucleotides, PCR primers, and small amplicons. Three independent paired-end libraries were sequenced on a HiSeq2000 system. The initial Illumina short reads from this study have been submitted to the NCBI sequence read archive (SRA, http://www.ncbi.nlm.nih.gov/Traces/sra/sra.cgi/) with the accession number SRA055764. 

### 2.3. *De Novo* Assembly of mRNA-Seq Reads, CDS Prediction, and Validation

Due to the fact that the base quality requirement for *de novo* assembly is more strict than that of the resequencing project, customized Perl scripts were used to remove reads which contained adaptor contamination, low quality bases (>2% base smaller than Q20 per read), and undetermined bases (>2% “N”s per read) from each dataset generated from different skin types. Three datasets were concatenated in a left-to-left and right-to-right manner. Next, the clean high-quality reads dataset was *de novo* assembled with default parameters by using Inchworm assembler which is a component of Trinity software [10]. All of the sequence reads were initially trimmed to 50 bp (nucleotides 21 to 70 of each read) in length and then used in mapping experiments and statistical analysis. Mapping short reads uniquely back to the contigs was performed by SOAPaligner 2.20 with two mismatched bases per read permitted [11]. Coding sequence prediction was performed by GENSCAN [12]. The contigs which contain two or three predicted CDSs may attribute to a small proportion of false positive discovery of predicting coding sequences from mature transcript sequences. Ten randomly selected putative full length transcripts which did not assign with known protein functions and another ten transcripts that are associated with hair cycling were subjected to perform reverse transcription polymerase chain reaction (RT-PCR) and Sanger sequencing. Primer sequences used for RT-PCR are available upon request. The assembled transcriptome sequences (49,115 contigs greater than 300 bp in length) in this study have been deposited to the NCBI's transcriptome shotgun assembly (TSA) database under the consecutive accession numbers from KA304519 to KA353633. 

### 2.4. Functional Annotation and Classification of Assembled Contigs

Mouse and Cow RefSeq protein sequences were downloaded from the NCBI RefSeq database (ftp://ftp.ncbi.nih.gov/refseq/). Homology searches against the Swiss-prot and NR databases were performed by using BLASTX algorithms with an *E*-value cutoff of 10^−10^. Mouse and Cow RefSeq RNA data were also downloaded as reference sequences for short reads mapping to calculate hit numbers compared with *de novo* assembled contigs. BLASTX was used to perform KOG and KEGG annotation [13, 14], followed by retrieval of the functional proteins and assignment to each of the classification entries (*E-*value 10^−10^). Gene ontology (GO) against the NR database was conducted by Blast2GO (*E-*value 10^−10^) [15]. WEGO [16] and GO terms classifications counter (http://www.animalgenome.org/tools/catego/) were used for assignment of each GO ID to the related ontology terms.

### 2.5. Digital Profiling of Differentially Expressed Transcripts and qRT-PCR Validation

According to the AC statistical framework [17], the *p* value of differential expression significance of each transcript-derived contigs between two samples was calculated by using the following equation:

(1) $$\begin{array}{c} p\left(y\;|\;x\right)={\left(\frac{N_{2}}{N_{1}}\right)}^{y}\frac{\left(x+y\right)!}{x!y!{\left(1+N_{2}/N_{1}\right)}^{\left(x+y+1\right)}}. \end{array}$$

*N*_1_ and *N*_2_ represent the total uniquely mapped reads in sample one and sample two, respectively. *x* is the number of reads mapped to a certain gene in sample one, and *y* represents the number of reads mapped to the same gene in sample two. After the calculation of *p* value, multiple hypothesis testing was performed to correct *p* value by using Phyper function of the R tool (http://www.r-project.org/). RPKM values of each contig were estimated by aligning trimmed reads back to the contigs [18]. Both a *q*-value of less than 10^−3^and a RPKM value with at least 2-fold difference between the two samples were used as criteria to determine significant DEGs. KOG enrichment analysis was conducted by hypergeometric distribution test by using the Phyper function in the R software package. Bonferroni correction was further used to adjust the *p* values, respectively. The significantly enriched functional clusters were selected when the corrected *p* value (*q*-value) was less than 10^−3^. Quantitative real-time PCR (qRT-PCR) was performed on the same individual of the corresponding body part skin. Total RNA was firstly treated with DNase I before reverse transcription by superscript III double-stranded cDNA synthesis kit (Invitrogen). Each cDNA sample was used as template for qRT-PCR by using the SYBR Premix Ex Taq II kit (TaKaRa) on a 7300 real-time PCR system (Applied Biosystems), and at least three technical repeats were performed for all the genes within each template. Acetyl-CoA carboxylase 1 (a7431; 102) was used to normalize gene expression quantities between samples. Gene expression fold difference between two samples was calculated by the 2^−ΔΔCt^ method [19]. PCR primer sequences are available upon request.

## 3. Results

### 3.1. Illumina Paired-End Sequencing, *De Novo* Transcriptome Assembly, and *Ab Initio *CDS Prediction

**Figure 1:**
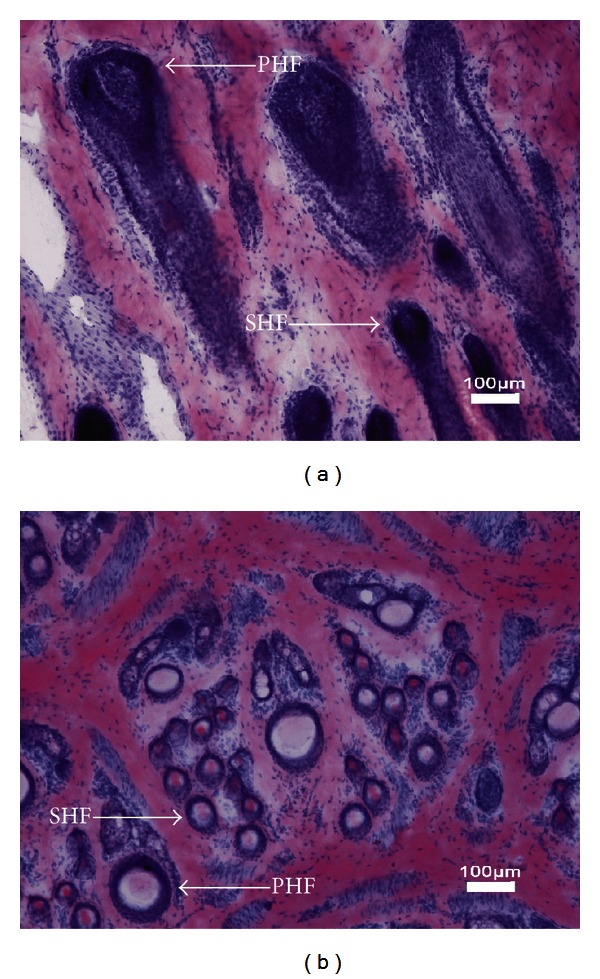
Frozen sections of cashmere goat skin stained by hematoxylin. White arrows indicate the primary hair follicles (PHFs) and secondary hair follicles (SHFs) in the sample.

To obtain comprehensive transcripts of skin tissue that provide an overview of gene expression profile during anagen in the cashmere goat, skin tissues from the belly (BL), back (BK), and the side of the body (BS) during anagen were sampled. The skin sections were made to show primary hair follicles and secondary hair follicles (Figure 1). Then, total RNA from each sample was isolated, respectively. Three RNA-Seq libraries were constructed and sequenced by using Illumina/Solexa technology. As a result, a total of approximately 130 million raw reads (65 million paired-end reads, 2 × 100 bp) that represented roughly 13 GB of sequence data were generated from three independent 200 bp insert libraries. The initial read quantities of three libraires were listed in Table 1. After the removal of reads with low quality and containing ambiguous bases (>2% “N” per reads), we employed Inchworm assembler to *de novo *assemble high quality reads which were generated from three different skin types. *De novo *assembly mRNA-Seq reads yielded 49,115 contigs over 300 bp comprising 45.4 MB of total sequence length, with an average length of 924 bp and N50 length of 1380 bp. Of the 49,115 contigs, there were 12,892 (26.2%) contigs greater than 1 Kb, 13,768 (28.1%) varying from 501 bp to 1 Kb, and the remaining 22,455 (45.7%) ranging from 301 bp to 500 bp in length (Table 2). To identify the protein encoding regions, we used GENSCAN to perform the *ab initio *prediction of the coding sequence (CDS) of 49,115 contigs. We found that 23,039 putative CDSs were identified from 22,734 (46.3%) assembled contigs. Of the 22,734 CDS-contained contigs, 22,440 have one putative CDS, 283 have two, and 11 contain three CDSs. Further analysis indicated 8,184 out of 23,039 contained a putative full-length CDSs (i.e., containing start and stop codons). Further, 6,889 CDSs contained a start but no stop codon, 3,171 predicted to have a stop but no start codon, and 4,795 have neither. The average length of putative 8,184 full-length CDSs reached 1,326 bp, while the partial CDSs have an average length of 605 bp. Among 8,184 predicted full-length CDSs, 127 of them cannot be annotated by known proteins. To validate sequence assemblies, we randomly selected ten predicted full-length CDSs that did not possess BLASTX hits in the NR database and ten genes which have been demonstrated that are specific to hair cycling and hair growth to perform RT-PCR and Sanger sequencing. These genes include the signaling molecules such as Wnt, insulin-like growth factor 1 (IGF-1), members of fibroblast growth factor (FGF), and their receptors encoding genes such as Frizzled and IGF-1R. The result showed that 19 PCRs are positive and the Sanger sequencing results all showed higher than 97% identities with *de novo* assembled transcripts indicating the relatively high credibility of sequences assemblies (Table S1 and Table S2 in Supplementary Material available online at http://dx.doi.org/10.1155/2013/269191). To further validate the quality and sequencing depth of the assembled contigs, we mapped the total short reads back to the assembled contigs. The sequencing depth ranged from 2- to 164,789-fold, with an average sequencing depth of 249-fold. Specifically, 87.4%, 87.5%, and 85.9% corresponding to the BL, BK, and BS skin of high-quality reads, respectively, can uniquely be realigned to the 49,115 contigs (Table 1). The relatively small proportion of unmapped reads may be involved in comprising the contigs which are shorter than 300 bp. This suggests that the majority of short reads in our RNA-Seq data were efficiently assembled into relatively larger contigs. 

### 3.2. Codon Usage and SSR Marker Identification

Examining the codon usage of 23,039 predicted CDSs showed that the most abundant amino acids encoded by triplet codons are nonpolar (hydrophobic) amino acids (44.1%), and then the uncharged polar amino acids (29.5%), while the acidic and basic amino acids accounted for 12.3% and 14.1%, respectively (Table S3). Among the 23,039 predicted CDSs, the average GC content reaches 54.9%, while the maximal GC content is 86.7% and minimum is 31.3%. Scanning the stop codon of 11,355 CDSs (8,184 predicted full lengths CDSs plus 3,171 stop codon containing CDSs) indicated that the stop codon most frequently used in goat is TGA which account for 53.8%, whereas TAG (23.2%) and TAA (23.0%) have the approximately equal utility frequency.

We used MISA (http://pgrc.ipk-gatersleben.de/misa/misa.html) to search the potential simple sequence repeats (SSRs) existed in our assembled transcripts. In this study, the repeat sequence which consists of dinucleotide, trinucleotide, tetranucleotide, pentanucleotide, and hexanucleotide tandem repeats with at least 18 bp in size was considered as an SSR. We found a total of 2,011 transcripts-derived SSRs that represent 158 unique repeat motifs scattered in 1,850 contigs of which 141 contigs contain at least 2 SSRs. The frequency of SSR occurrence is 4.09% and the average distance is 22.6 kb in our assembled 49,115 large contigs. 43.6% of the total SSRs are trinucleotide repeats, followed by dinucleotide (26.3%), hexanucleotide repeats (20.4%), and only a small proportion of them are pentanucleotide and tetranucleotide repeats (5.5% and 4.2%), respectively. The AC/GT (24.1%) motif comprises the highest frequency among all the identified SSRs, followed by CCG/CGG (18.4%), AGC/CTG (11.5%), and AGG/CCT (7.9%). The types of SSRs and their occurrence frequencies that we found in the goat are similar to the findings in other mammals but different from the results in plants. For example, the AC/GT repeat type, most abundant in the goat transcriptome sequence, is also very abundant in the human genome and in other vertebrates, whereas this repeat type is rarely observed in rice and sweet potato [20, 21]. The microsatellites identified in this work will become a valuable resource for goat genetic mapping. 

### 3.3. Functional Annotation of Transcript-Derived Contigs

**Figure 2:**
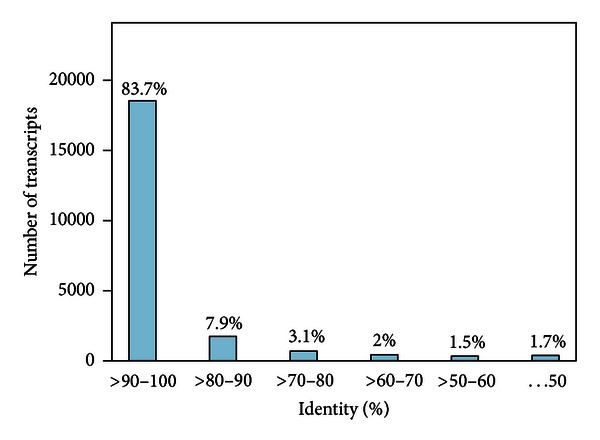
Sequence identity distribution. All BLASTX-hit transcripts were calculated. Vertical light blue bar shows the number of transcripts-derived contigs with which the range of percentage hit by BLASTX.

To functionally annotate the assembled contigs, a sequence similarity search was performed against *Bos taurus* RefSeq protein sequences (32,242 sequences), the Swiss-Prot protein database, and the nonredundant (NR) protein database by using BLASTX algorithm with a constant *E*-value (10^−10^). With this approach, 21,104, 21,193, and 22,146 contigs were annotated by *Bos taurus* RefSeq protein sequences, Swiss-Prot protein database, and the NR database, respectively. 20,984 out of 22,146 contigs showed ≥70% sequence identity with NR top hit at matched region (Figure 2). Among the 22,146 BLASTX-hit possessing contigs, it is worth to note that only 103 (0.47%) contigs corresponding to the NR database top hits match goat itself, which could explain the limited number of goat gene and protein sequences currently available in the public database. Examining the 22,146 (45.1%) contigs with a high similarity to NR, we found that 19,040 contigs also harbored a predicted CDS, demonstrating that many putative CDSs cannot be annotated by known functions and thus may indicate some new genes existed in our assembled contigs. The remaining 26,969 contigs which had no significant hits in the NR database are more likely 5′ and 3′-untranslated regions (UTRs) or previously uncharacterized ESTs (or genes specifically expressed in *Capra hircus*). These transcripts are shorter in average length and relatively less abundant in their sequencing depth compared with those contigs which significantly hit to the NR database (data not shown). However, we also noted that many contigs with high sequencing depth showed no hits with the NR database. In the top 1,000 most reads-abundant contigs, 69 contigs with an average length of 893 bp showed no hits with known proteins. In fact, when we aligned sequenced reads to the 36,442 *Mus musculus* and 34,573 *Bos taurus* RefSeq mRNA, only 8,717 (25.2%) and 14,836 (43.0%) sequences were mapped to each, respectively. This suggested that *Capra hircus* is not phylogenetically close to the other two mammals, even though *Capra hircus *belongs to the same family as *Bos taurus*.

Since hair (wool and cashmere) mainly consists of highly compressed dead keratinocytes, we elucidated the relative abundance by calculating the values of reads per kilobase per million reads (RPKM) of 49,115 contigs which enabled us examine the expression level of the keratin-encoding genes relative to other genes. Through BLAST searching, we found a total of 126 keratin or keratin-associated protein (KAP) encoding sequences presented in our contig database. As expected, of the total 49,115 contigs, the top ten most abundant exclusively encode keratin or KAP, including K5, K14, KAP3.1, K33B, and KAP1.1. The remaining 116 keratins or KAP associated contigs also showed a greater average abundance than other genes. We also noted that some of the keratin-related contigs exhibited relatively higher amino acid diversity (6 out of 126 with <80% identities and 11 with <90% identities at BLAST matched region) when compared with the NR database top hits, suggesting a series of novel keratin variants may be synthesized in skin tissue undergoing rapid hair (anagen phase) growth. The expression level of keratin or KAP may also give an insight into the promoter efficiency and selection while performing exogenous gene expression in the skin tissue. 

### 3.4. Functional Classification of Assembled Contigs by KOG, KEGG, and GO

We used BLASTX to search against functional proteins from the KOG (euKaryotic Orthologous Groups) database which is a component of the clusters of orthologous groups (COG) database [13]. 16,036 contigs had significant hits (*E*-value 10^−10^), and these were classified into 4 groups and 25 functional clusters. Apart from 4,750 poorly characterized genes, cellular process, and signaling appeared as the largest group, which consisted of three highly abundant clusters including signal transduction mechanisms (3,106 genes), intracellular trafficking, secretion, vesicular transport (906 genes), and cytoskeleton (864 genes). Furthermore, to analyze pathway-based biological activities of genes which were expressed in goat skin, we annotated the 49,115 contigs against the KEGG (http://www.genome.jp/kegg/) protein database. From our contig database, 15,020 (30.6%) contigs were assigned to the 291 KEGG pathways. Among them, 3,948 genes can be further assigned to 23 signaling pathways, including the MAPK, Wnt, Insulin, Hedgehog, TGF-beta, VEGF, and Notch pathways which have been previously demonstrated to play various important roles in HF development and hair shaft differentiation. 

**Figure 3:**
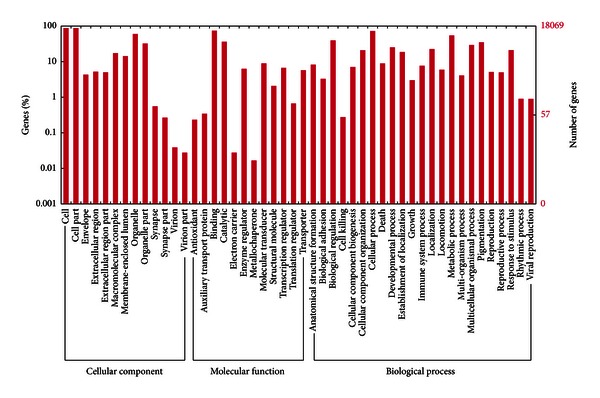
Gene Ontology classification of the transcriptome sequences. 18,069 transcriptome sequences can be annotated by the GO database. The classification results are displayed in the three main ontologies: cellular component, molecular function, and biological process.

In addition, gene ontology (GO) (http://www.geneontology.org/) analysis was also performed by using the Blast2GO program to further classify the transcript-derived contigs [15]. 18,069 contigs were cataloged into three main GO domains with a total of 129,669 GO IDs, and further subdivided into 47 subcategories (Figure 3). Of these assigned GO terms, biological process was the predominant domain followed by molecular function and cellular component. Under the biological process category, we found that cellular process and metabolic process are prominently represented, as they were in the KOG and KEGG classification, suggesting complicated metabolic activities occurred in anagen phase goat skin. A high correlation among KOG, KEGG, and GO classifications may reflect that goat hair growth is mediated by the complicated metabolic processes.

### 3.5. Differential Gene Expression Profiling, Functional Enrichment Analysis, and qRT-PCR Validation

**Figure 4:**
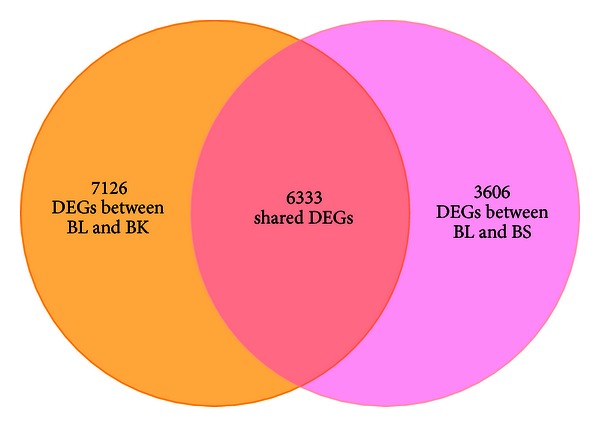
Venn diagram of shared DEGs between BL versus BK and BL versus BS comparisons.

A popular method of global measurement of differentially expressed genes is to take the quantity of NGS reads as an indicator for calculating transcript abundance [5–7]. Quantitative measurement of gene expression by NGS technologies has been suggested to be accurate and highly correlated with other methods of detecting gene expression levels, such as qRT-PCR and DNA microarray [5]. To identify differentially expressed genes (DEGs) among the three different skin types reflected in our short reads dataset, we mapped the short reads datasets from three libraries to the 49,115 contigs by SOAPaligner with the seed length of 50 bp. After mapping skin type-specific short reads to the reference, we calculated RPKM values for all contigs which can be used to quantify the expression of contigs both within and between samples [18]. An AC statistical framework was applied to calculate the *p* value of each transcript-derived contig expression difference by two-sample comparison [17]. Then, we performed multiple hypothesis testing by controlling the false discovery rate (FDR, *q*-value) to correct the *p-*value. In this study, both the FDR was less than 10^−3^ and the RPKM values were greater than 2-fold (or less than 1/2-fold) different between two samples, then they were considered as a statistically significant DEGs (Table S4). From BL-BK comparison, we observed that 3,532 transcript-derived contigs were upregulated expression from BK when compared to BL skin, and 9,927 were downregulated (Figure S1). Similarly, 3,128 upregulated and 6,811 downregulated contigs were detected in the comparison of BS skin with BL skin. Further analysis revealed that 1,360 transcript-derived contigs were consistently upregulated and 4,973 were downregulated in BK and BS compared to BL skin (Figure 4). However, the number of DEGs was sharply reduced to 5,367, of which 3,338 were upregulated and 2,029 were downregulated from BS to BK skin, indicating that the genes expression patterns between BK and BS are more similar than those two comparisons.

The BK and BS skin of the Cashmere goat mainly produce cashmere fiber, whereas BL skin mostly grows wool fiber but few cashmere fibers. Therefore, to investigate differences between these two kinds of skin types, we annotated the 6,333 consistent DEGs and further performed KOG enrichment analysis compared with transcriptome background. We found that the clusters involved in the cell cycle control, cell division, and chromosome partitioning in KOG classification were overrepresented by these DEGs (Table S5). The evidence is that 71 out of 1442 (4.92%) annotated DEGs (KOG database annotation) which derived from 538 counterparts of total 17,594 (2.97%) annotated transcriptome sequences (*p* < 8.47*e* − 6, *q*-value < 2*e* − 4). Furthermore, ten significant DEGs that enriched in this cluster were selected to perform qRT-PCR and to investigate gene expression difference among three skin types. Although the exact fold difference for each DEG by qRT-PCR were different from the RNA-Seq method but all the comparison pairs had the similar trends with RNA-Seq approach suggesting the relative high consistency between RNA-Seq and qRT-PCR (Table S6).

## 4. Discussion

To obtain the comprehensive transcripts of goat and its gene expression profiles reflected in different skin types during anagen phase, we sequenced and assembled mRNA from BL, BK, and BS skin of body coat. The assembler used in this work is Inchworm which is a major component of Trinity software [10]. Initially, the assembler generated 265,169 contigs over 100 bp (average contig length 299 bp and N50 length 417 bp) corresponding to ~79.3 MB sequence length, 87,962 contigs of which are longer than 200 bp (average contig length 622 bp and N50 length 1001 bp) representing ~54.8 MB nonredundant sequence length. When we use these smaller contigs for functional annotation, more redundant hit accessions were obtained, and the functional classification of transcripts-derived contigs would be more redundantly represented in each functional cluster, which gave us biased interpretation and overview of the transcripts functions. In this study, we used the contigs over 300 bp for annotation, which yielded 22,146 hits and represented 17,472 nonredundant accessions, indicating that the large proportion of the contigs belonged to UniGene clusters. On the other hand, approximately 87% of the total short reads can uniquely be mapped to the 49,115 contigs also suggesting that the majority of reads contributed to comprising those larger contigs.

As the different skin-regions of the Cashmere goat body coat, cashmere fibers are mainly produced from skin on the BK and BS, with few growing from the BL part. The molecules that are differentially expressed among these skin types may have underlying or potential roles associated with cashmere growth. Through calculating the short reads mapped on each contig from different libraries, we identified 6,333 consistently differentially expressed transcripts from BK and BS responded to BL skin (Figure 4). These DEGs were mainly enriched in the cell cycle control, cell division, and chromosome partitioning in the KOG functional clusters, indicating that the gene expression pattern associated with cell cycle and the cell division between two types of skin are significantly different. For instance, a kinetochore-bound protein kinase, named budding uninhibited by benzimidazoles 1 (Bub1), was identified 4- and 3-fold downregulation from BK and BS compared with BL skin (contig ID a91887;15). This kinase functions in part by phosphorylating a member of the miotic checkpoint complex and activating the spindle checkpoint [22, 23]. Similarly, we found another important kinetochore protein known as NDC80 (a132791;11) which is responsible for chromosomes segregation during M phase of the cell division that was also identified as a significant downregulated gene from BK and BS compared with BL skin [24]. In addition to the genes involved in the spindle checkpoint during cell division, we also noted that some important regulators which directly participate in the regulation of cell cycle progress are differentially expressed. For example, we found that cyclin-dependent kinase 7 (CDK7, a53471;29) as well as its partner, Cyclin-H (a65131;18), which form a complex to directly regulate cycle division were both identified as downregulated DEGs in BK and BS libraries compared within BL part [25]. Furthermore, profiling of cell cycle associated DEGs by using qRT-PCR method also showed relativelyhigh consistent result with RNA-Seq. This may suggest that the hair synthesis rates between cashmere- and wool-producing skins are significantly different.

A large body of literature has focused on revealing the molecular mechanisms of HF initiation, patterning, and hair cycling in mammalian model organisms such as *Mus musculus*. Many significant studies mainly focused on the function of upstream molecules of the signal transduction pathway such as Wnt/*β*-catenin, TGF-*β*, Eda, Hedgehog, IGF, and their receptors [26–42]. Generally, conditional knockout or skin tissue-specific overexpression of ligand, receptor, or adaptor molecules from these pathways during the embryogenesis or postnatal stages usually has diverse effects on HF and hair shaft formation. One example is *β*-catenin which serves as an important adaptor molecule in embryogenesis. Sustained epithelial *β*-catenin activation by a transgenic approach caused excessive induction and fusion of HF with severely impaired fiber shaft formation [43]. However, the factors which directly promote hair growth are rarely characterized. The most compelling molecule discovered to promote hair growth is FGF-5, a secreted protein that when ablated from mice leads to abnormally long hair (~1.5-fold longer than wild type) by either the elongation of the anagen phase or retardation of catagen initiation [44]. IGF-1, another important mitogen associated with HF development, has been reported as an elongation factor of mouse whiskers [31] and was recently demonstrated to promote body hair growth by overexpression of IGF-1 in mouse skin through transgenic approach [30]. But this growth was accompanied by an absence of two types of body coat hair and disorientation of a small proportion of HF [30]. Nevertheless, we did not identify that these molecules encoding genes were significantly differentially expressed. This may ascribe to the three samples that were all derived from anagen phase of the hair cycle, because many previous studies have demonstrated that these signaling molecules and their receptors function as important regulators during the transition from telogen (resting phase) to anagen, such as Wnt, Sonic hedgehog, and TGF-*β* family members [45–47], suggesting that the expression levels of these signals are different between two periods. In our DEG catalogue, only the genes associated with cell division and cell cycle are significantly enriched, which might indicate that the efficiency of hair synthesis is different between two skin types. The function of these enriched DEGs involved in the hair cycling should be further characterized.

## 5. Conclusions

Taking into consideration the read abundance, average contig length, N50 length, and total contig size, our assembled contig catalogue provided a relatively complete and comprehensive dataset which could reflect the goat skin transcriptome during the anagen phase. The identification of numerous genes, including those showing differential expression in three skin types, especially those DEGs which are enriched in the functional cluster, will provide us good launching points and resources for further characterizing gene functions associated with hair growth. Our dataset was generated solely with the use of an Illumina HiSeq2000 platform, demonstrating that this ultra-high-throughput sequencing technology is a suitable tool for investigation of the large eukaryotic organism transcriptome and global measurement of gene expression profile. Finally, the extremely abundant paired-end reads generated from anagen phase will be very useful for subsequent studies, such as comparisons with gene expression patterns from catagen or telogen phase, which will be very helpful for further identifying genes associated with hair follicle development and fiber growth. 

## Supplementary Material

Figure 1: Schematic representations of the gene expression profile in three goat skin libraries. (A) Transcript abundance levels in the belly and back skin library (BL vs BK). (B)Transcript abundance levels in the belly and side of body skin library (BL vs BS). (C) Transcript abundance levels in the back and side of body skin library (BK vs BS).
Table 1: Validation of ten putative full-length CDSs was conducted by using RT-PCR and Sanger sequencing.
Table 2: Validation of ten transcripts which were previously demonstrated specific to hair cycling by using RT-PCR and assembly annotation.
Table 3: Codon usage of 23,039 predicted CDSs of the Cashmere goat.
Table 4: Functional annotation and gene expression patterns from BL, BK and BS skin RNA-Seq libraries of the Cashmere goat during anagen phase.
Table 5: KOG enrichment analysis of 6333 consistently differentially expressed genes compared with transcriptome background.
Table 6: Examining differentially expressed genes by using qRT-PCR and direct comparison with RNA-Seq.

## Figures and Tables

**Table 1:** Read number and mapping result from three independent libraries.

Sample	Total reads	Clean reads	Mapped reads	Mapped/clean
BL	41232601	40731588	35593595	87.4%
BK	39363238	38876298	34033557	87.5%
BS	49962602	49337308	42383188	85.9%

**Table 2:** Length distribution of 49,115 assembled contigs.

Contig length (bp)	Number of contigs	Cumulative size
301 ~ 500	22455	8.5 Mb
501 ~ 700	7987	4.7 Mb
701 ~ 1000	5781	4.8 Mb
1001 ~ 1500	4908	6.0 Mb
1500 ~ 2000	2914	5.1 Mb
>2000	5070	16.3 Mb

Total	49115	45.4 Mb
