# Supplementary figures and images for: De Novo Transcriptome Assembly and Differential Gene Expression Profiling of Three Capra hircus Skin Types during Anagen of the Hair Growth Cycle
Source: Int J Genomics. 2013 May 20;2013:269191. doi: 10.1155/2013/269191 (PMC3671518; doi:10.1155/2013/269191)

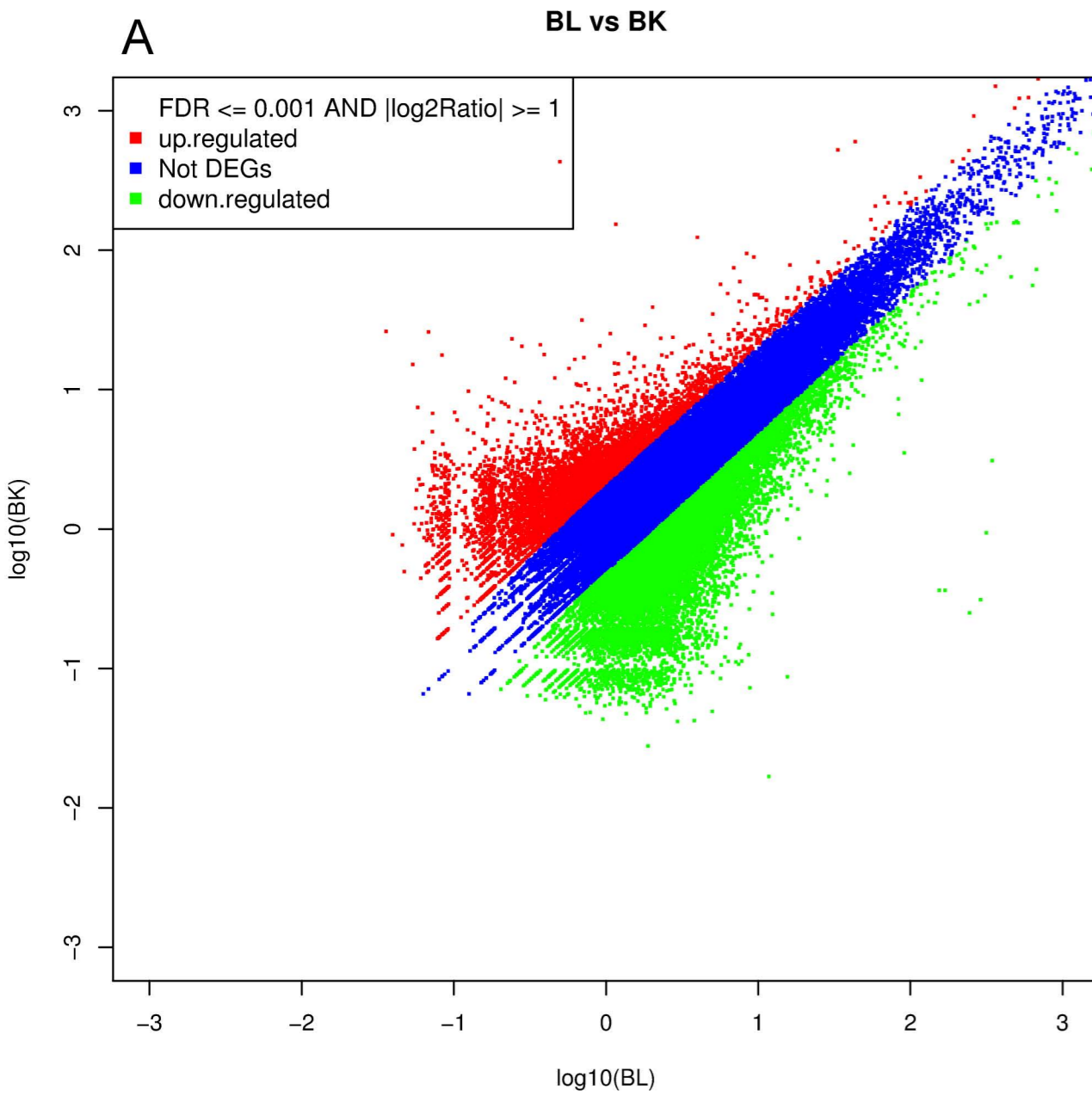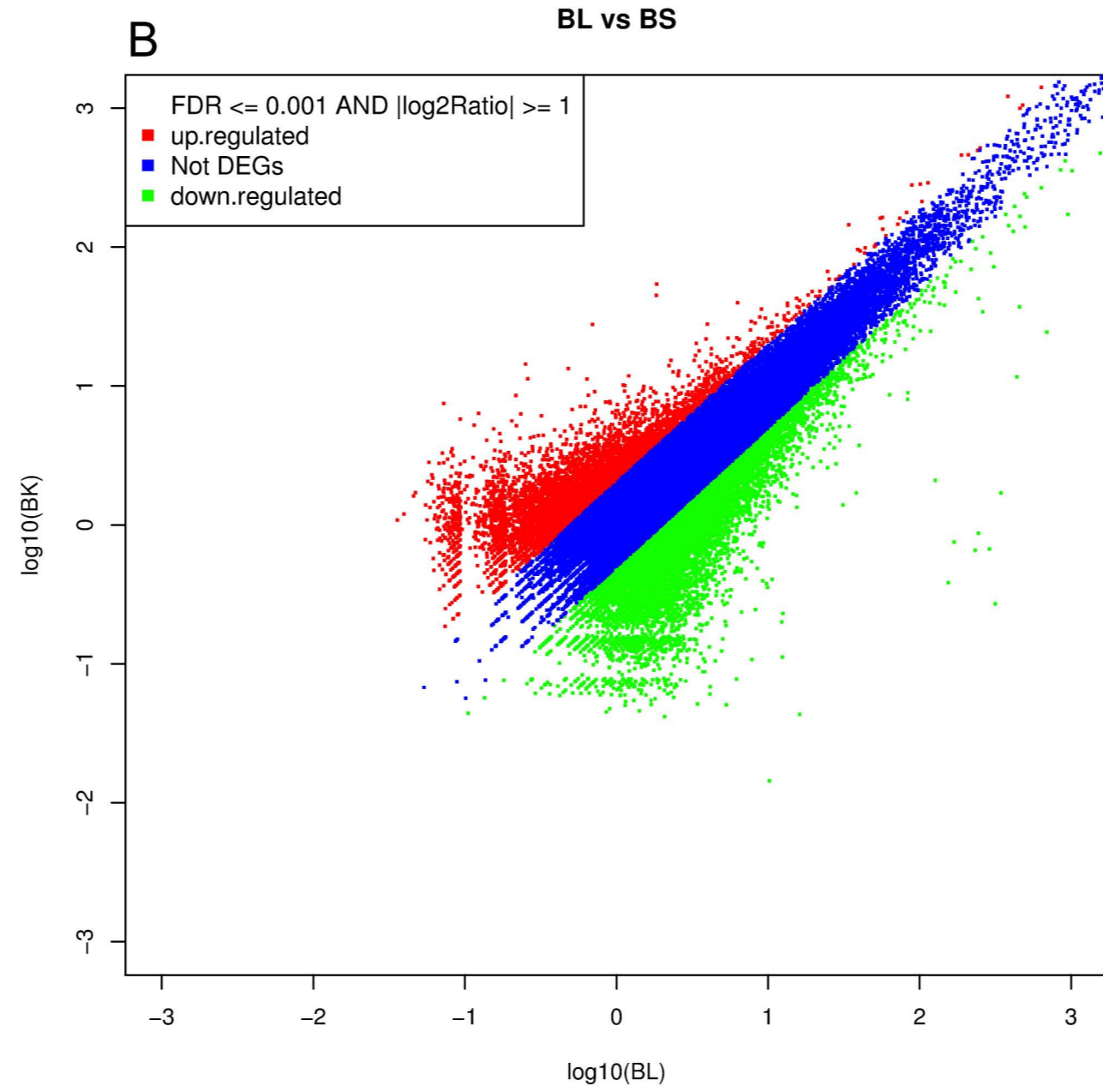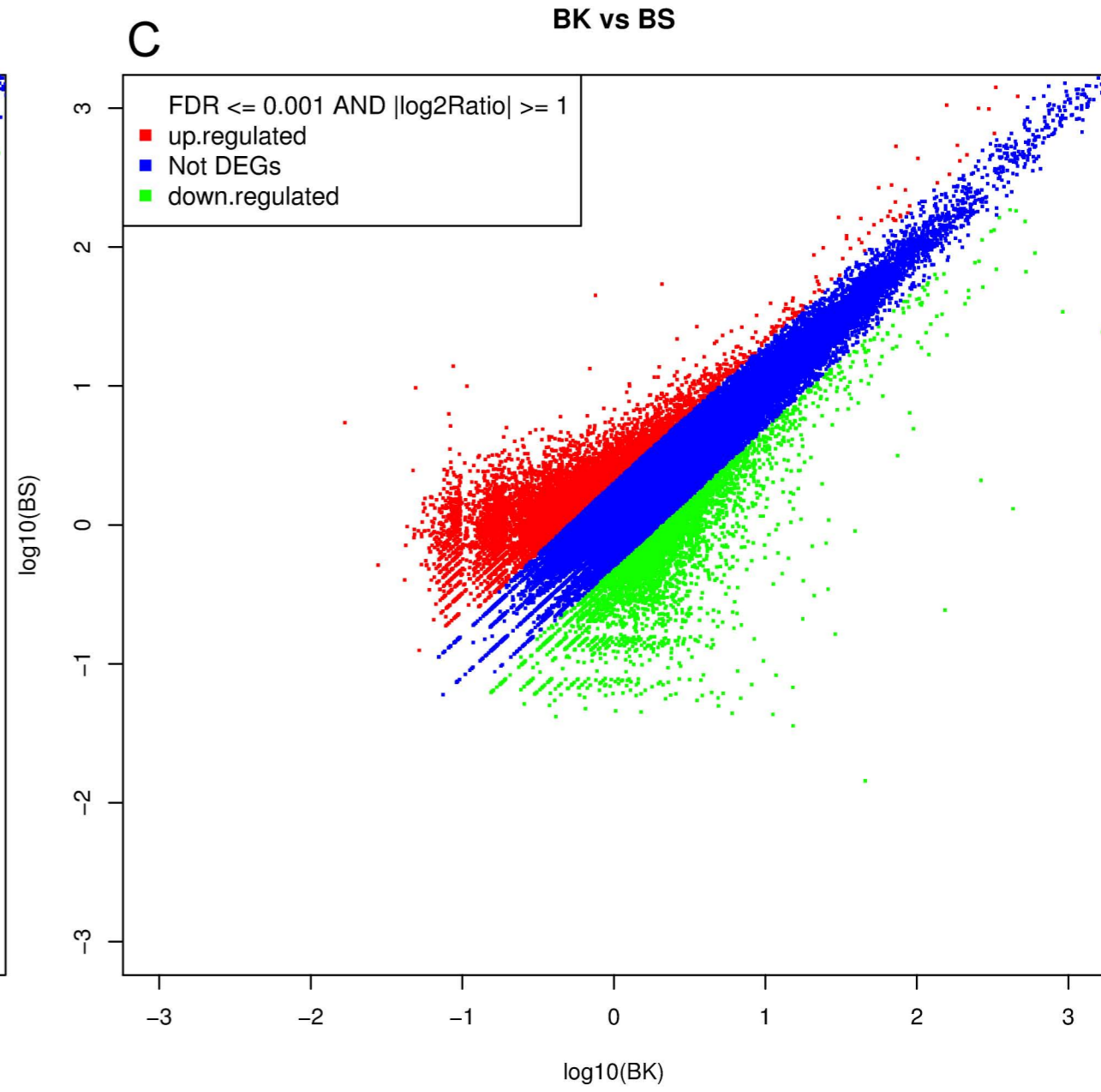

Supplement: Supplementary file 1 — Figure 1: Schematic representations of the gene expression profile in three goat skin libraries. (A) Transcript abundance levels in the belly and back skin library (BL vs BK). (B)Transcript abundance levels in the belly and side of body skin library (BL vs BS). (C) Transcript abundance levels in the back and side of body skin library (BK vs BS). Table 1: Validation of ten putative full-length CDSs was conducted by using RT-PCR and Sanger sequencing. Table 2: Validation of ten transcripts which were previously demonstrated specific to hair cycling by using RT-PCR and assembly annotation. Table 3: Codon usage of 23,039 predicted CDSs of the Cashmere goat. Table 4: Functional annotation and gene expression patterns from BL, BK and BS skin RNA-Seq libraries of the Cashmere goat during anagen phase. Table 5: KOG enrichment analysis of 6333 consistently differentially expressed genes compared with transcriptome background. Table 6: Examining differentially expressed genes by using qRT-PCR and direct comparison with RNA-Seq. [file 269191.f1.pdf]
